# 

**Published:** 1882-07

**Authors:** 


### Pamphlets.

Report of the Medical Director of the Citizens' Sanitary Association of Savannah, Ga. By J. C. LeHardy, M.D.

The Diagnosis of Pott's Disease of the Spine, before the stage of deformity. By V. P. Gibney, A.M., M.D. Reprint from *Boston Medical and Surgical Journal.*

Genius Resistless, an ode. A tribute to Jenner and Pasteur. By J. J. Caldwell, M.D., "Neurologist" ! ! !

A contribution to the subject of nerve-stretching. By

William J. Morton, M.D. Reprint from the *Journal of Nervous and Mental Disease.*

The Worcester Sewage and the Blackstone River.

The Estimation of Urea by Sodium Hypobromite. By J. R. Duggan, M.D. Reprint from the *American Chemical Journal.*

